# Time for sex: nycthemeral distribution of human sexual behavior

**DOI:** 10.1186/1740-3391-3-4

**Published:** 2005-03-24

**Authors:** Roberto Refinetti

**Affiliations:** 1 Circadian Rhythm Laboratory, University of South Carolina, 807 Hampton Street, Walterboro, SC 29488, USA

## Abstract

**Background:**

Nycthemeral (daily) oscillation has been documented in a variety of physiological and behavioral processes. The present study was carried out to evaluate the existence of a nycthemeral rhythm of human sexual behavior and to identify environmental factors responsible for the rhythmic pattern.

**Methods:**

Non-traditional university students (ages 18 to 51 years) recorded the times of day when they went to sleep, when they woke up, and when they had sex for 3 consecutive weeks. They also answered a questionnaire designed to identify the causes of their selection of time for sex.

**Results:**

The majority of sexual encounters took place at bedtime (11 pm to 1 am). The most common explanations for this temporal pattern were the rigidity of the professional work schedule and family obligations and the availability of the partner, which reduced the opportunity for sexual encounters at other times of the day.

**Conclusion:**

Most sexual encounters take place around bedtime. Although the presence of an endogenous component responsible for this temporal pattern cannot be excluded, the evidence indicates strong environmental forcing, particularly from the work/family schedule of the individuals and from partner availability.

## Background

Practically all physiological parameters in the animal and human body exhibit nycthemeral (daily) or circadian oscillation [1,2]. Epidemiological studies have documented nycthemeral oscillation in a variety of aggregate variables, such as heart attacks [3-5], births [6-8], and suicides [9-11]. One study conducted in 1982 provided evidence of the existence of a nycthemeral rhythm of sexual activity in young married couples [12]. The present study sought to verify this nycthemeral rhythmicity in a sample of human adults with a wider age range and to identify environmental factors responsible for the rhythmic pattern.

## Methods

### Part 1

The first part of the study, conducted during the winter of 2003, involved 15 non-traditional university students in South Carolina (6 males, 9 females, ages 18 to 51 years). The subjects participated in the study as partial fulfillment of course requirements. They were given a data sheet and were asked to record, each day for three weeks, the times of day when they went to sleep, when they woke up, and when they had sex. Subjects were allowed to use their own definition of sex, which did not necessarily involve vaginal intercourse. Complete forms were received from 11 subjects, anonymously. All subjects were married or were involved in a steady relationship with a partner.

The accumulated nycthemeral distribution of sexual encounters was analyzed in three ways. A Kolmogorov-Smirnov test [13] was used to determine whether the experimental distribution differed significantly from a flat distribution. A Rayleigh test [14] was used to determine whether the experimental distribution conformed to a sinusoidal pattern. A cosinor test [15] was used to determine the acrophase (time of peak) of the nycthemeral oscillation.

### Part 2

The second part of the study, conducted during the winter of 2005, involved a separate group of 38 university students (14 males, 24 females, ages 18 to 43 years). The subjects were given a brief survey containing two main questions: "What time of day do you usually have sex?" and "Why do you have sex at these times (as opposed to other times of day)?". Answers could be written in for both questions, but potential answers were provided for the second question, as follows:

__ I feel more sexual at these times.

__ These are the only times when my partner is available.

__ My work/family schedule does not allow me to have sex at other times.

__ I'm already in bed, so why not have sex?

__ Other. Specify: ________________________________

Ten subjects (4 males, 6 females, aged between 18 and 20 years) had never had sex and, therefore, were excluded from the study. The percentages of the answers to the second question were computed and compared by the Kolmogorov-Smirnov test.

## Results and Discussion

### Part 1

Although there were not enough data for the analysis of the distribution of sexual encounters according to day of the week, the mean number of sexual encounters per week (2.3) was comparable to that found in the broader adult population of the United States (twice a week) [16].

**Figure 1:**
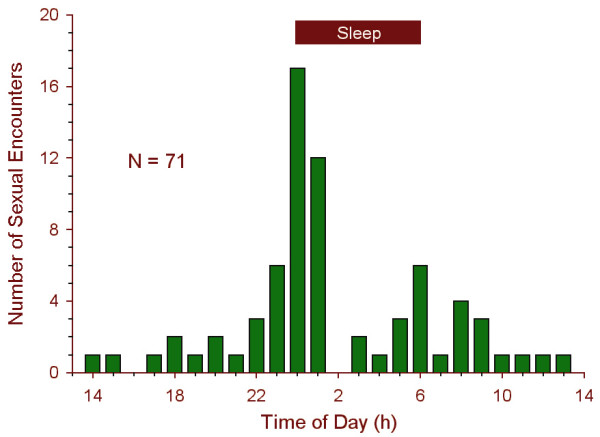
**Nycthemeral distribution of sexual encounters. **Of 71 recorded encounters, 17 took place at midnight, which was the average to-bed time for the subjects.

The nycthemeral distribution of sexual encounters is shown in Figure 1. Of 71 recorded encounters, 17 took place at midnight, which was the average to-bed time for the subjects. A smaller peak occurred at 06:00, which was the average wake-up time for the subjects. The Kolmogorov-Smirnov test indicated that the distribution was significantly different from a flat distribution (*D *= 0.201, *p *< 0.01), and the Rayleigh test indicated that the distribution conformed to a sinusoidal pattern (*nR*^2 ^= 15.706, χ^2^(2) = 31.412, *p *< 0.0001). The cosinor test indicated an acrophase at 01:00.

The mean bedtime of 00:00 and mean waketime of 06:00 are within the range of bedtimes and waketimes observed in various societies around the world [17-23]. The observed bedtime peak of sexual encounters is in agreement with a previous study of young married couples [12]. The results are also in agreement with those obtained in a survey about the time of day of the first intercourse of young women, in which it was found that over 85% of the subjects had lost their virginity either in the evening or at night [24].

### Part 2

Because the subjects in Part2 were asked only to recall their usual time of sex, the precision of the results was lower than in Part 1. The answers were categorized as "morning" (20%), "afternoon" (5%), "evening" (10%), and "night" (65%). This distribution is significantly different from a flat distribution (*D *= 0.400, *p *< 0.001) and is consistent with the results from Part 1.

**Figure 2:**
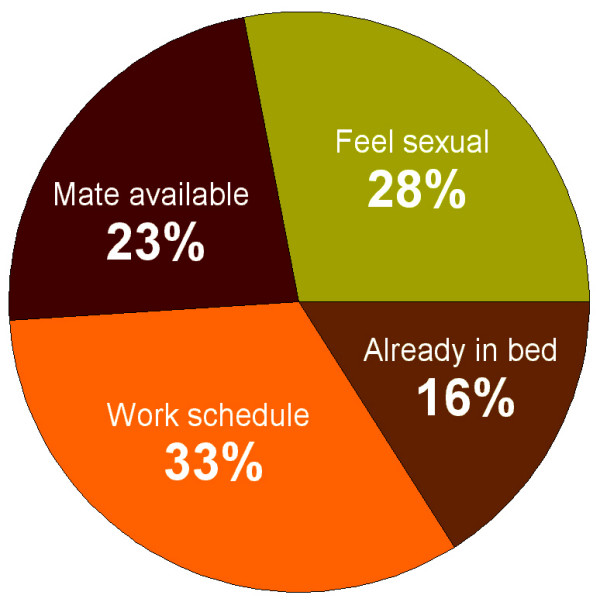
**Frequency distribution of answers to the question "Why do you have sex at these times?" **Most subjects indicated that the selection of time for sex is determined by external factors, such as work schedule and partner availability.

The distribution of responses to the question "Why do you have sex at these times?" is shown in Figure 2. Although the subjects were allowed to give multiple answers, most of them gave only one answer, and the few who chose "Other" actually wrote in one the four offered options. Consequently, the percentages of answers conveniently added up to 100%. Only 28% of the respondents gave an answer that would characterize an endogenous rhythm of sexual appetite ("I feel more sexual at these times"). In contrast, 33% of the respondents attributed the time selection to restrictions imposed by work/family schedule, and 23% attributed it to partner availability. The distribution of percentages does not differ significantly from a flat distribution (*D *= 0.090, *p *> 0.05), which indicates that none of the four responses was consistently chosen more frequently than the others. Thus, the response based on an endogenous rhythm of sexual appetite was chosen by only a quarter of the respondents. Even this relatively small fraction may be an overestimate, as the subjects may have failed to identify environmental factors other than those offered as potential answers.

In addressing the issue of exogenous causation, it must be emphasized that this study was conducted under normal living conditions, where environmental factors can "mask" the expression of an endogenous rhythm of sexual appetite. In men, it is known that there is daily rhythmicity in plasma concentration of testosterone [25,26], which likely has an endogenous source and could be responsible for rhythmicity in sexual appetite. However, the results of this study clearly indicate that, if there is an endogenous rhythm of sexual appetite, it is not the main determinant of the selection of time for sex in individuals living under normal societal conditions. Instead, the time for sex seems to be determined predominantly by environmental forcing.

## Conclusion

Although human adults seem to find opportunities for sex at practically any time of the day, most sexual encounters occur around bedtime (11 pm to 1 am). A smaller peak in sexual activity occurs around wake time. Because the study was conducted in the presence of external time cues, the issue of the endogenous source of this nycthemeral variation cannot be directly addressed. However, self-reports indicate the presence of strong environmental forcing, particularly from the work/family schedule of the individuals and from partner availability.

## Competing Interests

The author(s) declare that they have no competing interests.
